# Prognostic Value of C-reactive Protein and Neutrophil-to-Lymphocyte Ratio in Predicting Postoperative Infections After Gastrointestinal Surgery: A Meta-Analysis

**DOI:** 10.7759/cureus.91123

**Published:** 2025-08-27

**Authors:** Wishal Shaukat, Abdul Moeed Baig, Zulfiqar Ali, Komal Kumari, Talha Tariq, Abdul Karim Soomro, Adonia Flemming, Muhammad Tariq Hamayun Khan

**Affiliations:** 1 General Surgery, University Hospitals Birmingham, Birmingham, GBR; 2 Medicine, Gujranwala Medical College, Gujranwala, PAK; 3 General Surgery, Rashid Latif Medical College, Lahore, PAK; 4 Internal Medicine, Ziauddin University, Karachi, PAK; 5 Internal Medicine, Rahim Yar Khan (RYK) Teaching Hospital, Rahim Yar Khan, PAK; 6 Pathology, Bilawal Medical College, Jamshoro, PAK; 7 Gastroenterology, Washington University of Health and Science, San Pedro, BLZ; 8 Pathology, Burns and Plastic Surgery Centre Hayatabad Peshawar, Peshawar, PAK

**Keywords:** c-reactive protein, gastrointestinal surgery, neutrophil-to-lymphocyte ratio, postoperative infections, prognostic biomarkers

## Abstract

Postoperative infectious complications remain a significant cause of morbidity following gastrointestinal (GI) surgeries, necessitating early and reliable prognostic markers to guide timely intervention. This meta-analysis aimed to evaluate the predictive value of two widely available inflammatory biomarkers, C-reactive protein (CRP) and neutrophil-to-lymphocyte ratio (NLR), in forecasting postoperative infections in patients undergoing GI surgery. A systematic search of databases and trial registries was conducted, and eight studies meeting the inclusion criteria were analyzed. A total of 2,020 patients were included across these studies, covering various GI surgical procedures such as gastric resection, bile duct repair, appendectomy, and Crohn’s disease surgery. The meta-analysis revealed that elevated NLR was significantly associated with an increased risk of postoperative infections, with a pooled odds ratio (OR) of 2.54 (95% CI: 1.84-3.52, p < 0.00001) and moderate heterogeneity (I² = 38.6%). Likewise, CRP and the CRP-to-albumin ratio (CAR) also demonstrated strong predictive value, with a pooled OR of 2.43 (95% CI: 1.45-4.09, p = 0.0007) and low heterogeneity (I² = 22.4%). These findings are in line with prior research that supports the role of systemic inflammatory markers in perioperative risk assessment. The use of NLR and CRP-based markers offers a simple, cost-effective, and accessible approach for early identification of patients at high risk for infectious complications. This can help clinicians initiate preventive strategies and optimize postoperative care. While further large-scale, prospective studies are needed to standardize cutoff values and timing of assessment, our results support incorporating NLR and CRP into routine perioperative evaluation in GI surgical settings.

## Introduction and background

Postoperative infections remain a significant source of morbidity, mortality, and prolonged hospitalization in patients undergoing gastrointestinal (GI) surgery [1]. Despite advances in surgical techniques, perioperative care, and infection prevention strategies, the incidence of infectious complications such as surgical site infections (SSIs), intra-abdominal abscesses, pneumonia, and anastomotic leakage continues to pose a major clinical challenge [2,3]. An observational study over a seven-year period reported that the prevalence of postoperative infections ranged between 0.5% and 1.6%, with a rising trend in organ/space SSIs from 1.1% to 1.5% [4]. These complications not only increase healthcare costs but also compromise patient recovery and survival.

Currently, there is no universally accepted gold standard for the early detection of postoperative infectious complications. Traditional diagnostic tools, including microbiological cultures and clinical signs, often fail to provide timely or reliable information. Cultures may have low positivity rates and require time to yield results, while clinical symptoms typically manifest after the infection has progressed [5]. As such, there is a critical need for accessible, cost-effective, and sensitive biomarkers that can aid in the early risk stratification and prediction of postoperative infections.

C-reactive protein (CRP), an acute-phase reactant synthesized by the liver in response to systemic inflammation, has been widely studied for its diagnostic and prognostic utility in surgical settings [6]. Elevated postoperative CRP levels, particularly on days three to five after surgery, have been associated with intra-abdominal septic complications such as anastomotic leakage following colorectal resections [7]. However, CRP levels alone may not always offer sufficient sensitivity or specificity for predicting adverse outcomes across diverse surgical populations [8].

In this context, hematological inflammatory markers derived from routine complete blood counts (CBC), such as the neutrophil-to-lymphocyte ratio (NLR), have garnered increasing interest. NLR, calculated as the ratio of absolute neutrophil count to lymphocyte count, reflects the balance between pro-inflammatory and immune-regulatory processes. It has been proposed as a cost-effective and rapidly obtainable indicator of systemic inflammation, with studies suggesting its prognostic value in conditions ranging from cardiovascular disease to malignancy and acute infection [9,10].

In surgical cohorts, elevated preoperative NLR has been linked to poor outcomes, including increased risk of infectious complications and postoperative mortality [11]. Specifically, in GI surgery, preoperative NLR has shown promise in predicting 30-day morbidity and has been correlated with the development of serious complications such as anastomotic leakage after colorectal resections [12]. Notably, NLR appears to respond more rapidly to inflammatory changes than CRP, making it a potentially useful tool in the early postoperative period [13].

Despite growing evidence, the predictive accuracy of CRP and NLR for postoperative infections in GI surgery remains uncertain. Existing studies report heterogeneous findings and are often limited by small sample sizes, varying surgical populations, and inconsistent timing of biomarker assessment [12,14]. Moreover, there is a lack of comprehensive synthesis of the available data to determine the true prognostic value of these markers in clinical practice.

Therefore, this meta-analysis aims to systematically evaluate the existing literature to determine the prognostic performance of CRP and NLR in predicting postoperative infectious complications following GI surgery. By integrating data across multiple studies, this review seeks to provide evidence-based insights that can inform clinical decision-making, guide perioperative risk stratification, and potentially improve postoperative outcomes.

## Review

Materials and methods

Search Strategy

This meta-analysis was conducted in accordance with the Preferred Reporting Items for Systematic Reviews and Meta-Analyses (PRISMA) guidelines [15]. A comprehensive literature search was performed across PubMed, Scopus, Web of Science, Cochrane Central Register of Controlled Trials (CENTRAL), and Google Scholar for studies evaluating the predictive value of CRP and NLR for postoperative infections following GI surgery. The search covered articles published between January 2021 and July 2025 and was limited to studies published in English. The search terms used included combinations of Medical Subject Headings (MeSH) and keywords such as “C-reactive protein,” “neutrophil-to-lymphocyte ratio,” “postoperative infection,” “gastrointestinal surgery,” and “predictive biomarkers.” All retrieved records were imported into EndNote X9 (Clarivate Analytics, Philadelphia, PA, USA) to remove duplicates prior to screening.

Study Selection

Two independent reviewers screened the titles and abstracts of retrieved articles for eligibility. Full texts of potentially relevant studies were assessed. Disagreements were resolved through discussion and consensus. Inclusion criteria were adult patients undergoing GI surgery; studies assessing CRP and/or NLR levels in relation to postoperative infectious complications; availability of data on sensitivity, specificity, or sufficient data to calculate diagnostic accuracy; prospective or retrospective observational studies, and randomized controlled trials. Exclusion criteria included pediatric populations; case reports, reviews, editorials, and conference abstracts; and studies without sufficient outcome data.

Data Extraction

Data extraction was performed independently by two reviewers using a standardized form. Extracted information included first author, year of publication, country, study design, sample size, type of GI surgery, timing and thresholds of CRP/NLR measurements, and outcomes related to postoperative infections. Sensitivity, specificity, positive predictive value (PPV), negative predictive value (NPV), and area under the receiver operating characteristic curve (AUC) were collected or calculated when not directly reported.

Statistical Analysis

Meta-analysis was conducted using R Studio (version 2022.02.0-443, RStudio PBC, Boston, MA, USA) with the 'meta' and 'mada' packages. Pooled estimates of sensitivity, specificity, and diagnostic odds ratios (DORs) were calculated using a bivariate random-effects model [16]. Summary receiver operating characteristic (SROC) curves were generated, and the AUC was calculated to assess overall diagnostic performance. Heterogeneity was assessed using the I² statistic and the chi-squared test. Sensitivity analyses were performed to evaluate the robustness of the results.

Summary of the included studies

**Figure 1:**
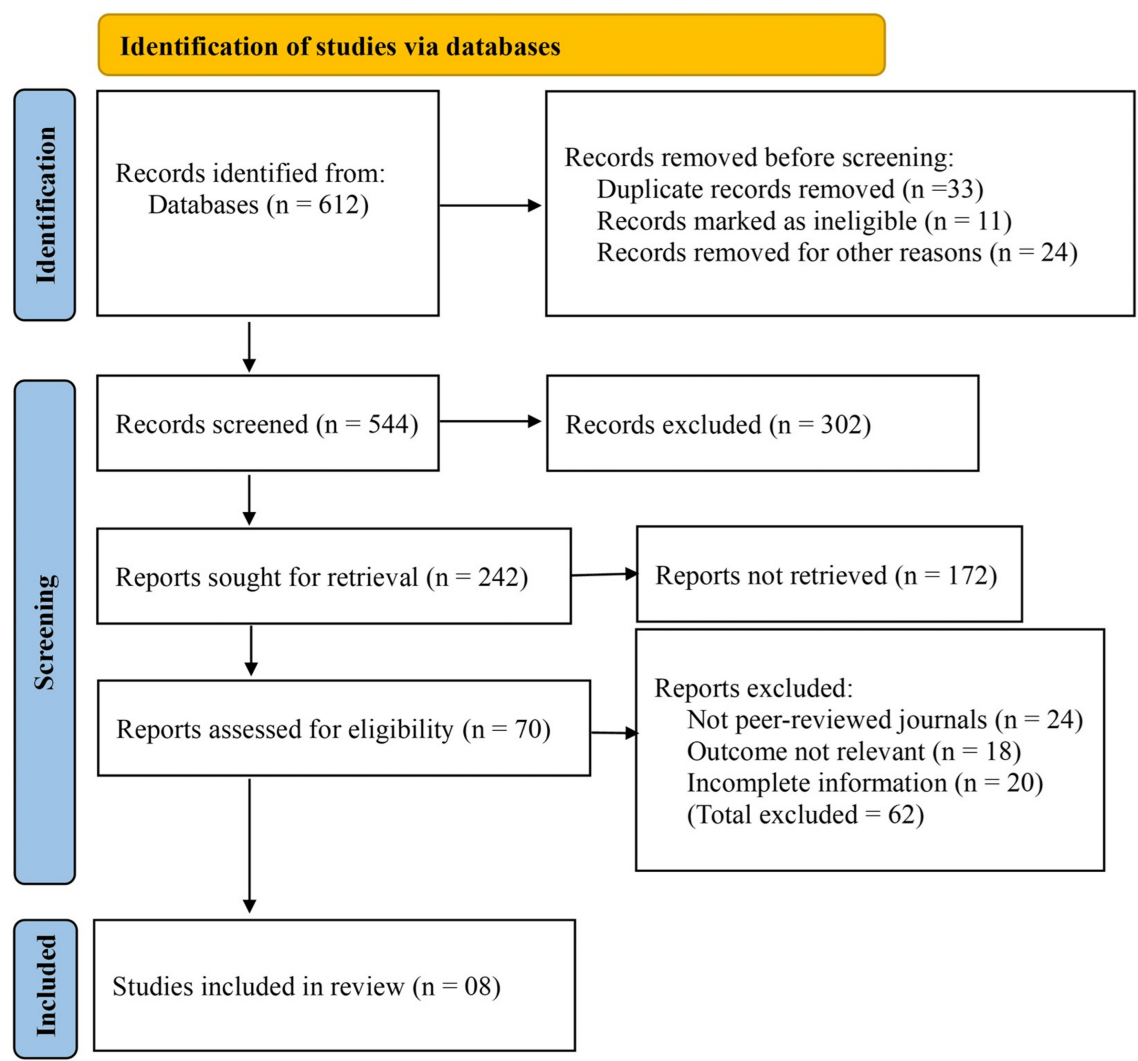
Preferred Reporting Items for Systematic Reviews and Meta-Analyses (PRISMA) flow diagram of the study selection process

A total of 612 records were identified through database searches. Before screening, 33 duplicate records were removed, along with 11 records marked as ineligible by automated tools and 24 records excluded for other reasons, such as conference abstracts or language limitations. This left 544 records for title and abstract screening, of which 302 were excluded due to irrelevance to the study objectives or insufficient methodological quality. Subsequently, 242 reports were sought for full-text retrieval, but 172 could not be accessed due to availability issues. The remaining 70 full-text articles were assessed for eligibility. Of these, 24 were excluded for being non-peer-reviewed publications, 18 lacked outcomes relevant to postoperative infections, and 20 provided incomplete or unclear data on CRP or NLR values. Ultimately, eight studies met the inclusion criteria and were incorporated into the final meta-analysis (Figure 1).

The final meta-analysis included eight studies that investigated the prognostic value of CRP and/or the NLR in predicting postoperative infectious complications following various types of GI surgery. The studies were conducted in diverse geographical regions, including Spain, Turkey, Mexico, Japan, and Israel, and encompassed a total sample size of 2,020 patients.

Study methodologies varied, including both prospective and retrospective cohort designs, ensuring a mix of high-quality data and real-world clinical insights. Sample sizes ranged widely, from 50 patients in a focused bile duct injury repair study to 1,136 pediatric patients undergoing appendectomy, allowing for generalizability across different GI surgical populations [17-24].

NLR emerged as a consistently valuable biomarker across nearly all included studies. For instance, Ortiz-López et al. [17] demonstrated that a high NLR on postoperative day 1 significantly predicted major complications after gastric oncologic surgery, whereas CRP did not show statistical significance in the same cohort. Similarly, in a large-scale pediatric appendectomy cohort, Delgado-Miguel et al. [18] found NLR to be the strongest predictor of SSI, with an AUC of 0.808 and strong sensitivity and specificity at a cutoff value of 9.8.

In Crohn’s disease surgical populations, both Kawamoto et al. [19] and Mullin et al. [20] identified elevated preoperative NLR as an independent predictor of intra-abdominal septic complications and major postoperative outcomes, respectively. These findings underline NLR’s relevance in inflammatory bowel disease-related surgeries.

CRP, while also showing predictive utility, appeared to be more context-dependent. For instance, Martinez-Mier et al. [21] and Evirgen & Cetin [22] reported statistically significant associations between elevated preoperative CRP levels and postoperative complications, particularly when combined with other markers like albumin to compute the CRP-to-albumin ratio (CAR).

Notably, the study by Evirgen & Cetin [22] provided additional depth by comparing CAR, NLR, and PLR in patients undergoing percutaneous endoscopic gastrostomy (PEG), identifying CAR as the most robust predictor in multivariate analysis. The study by Pantoja Pachajoa et al. [23] conducted in Argentina evaluated the prognostic performance of CRP and NLR for predicting anastomotic leakage following colorectal surgery. Among 116 patients, CRP measured on postoperative day 5 showed superior diagnostic accuracy, with an AUC of 0.81, sensitivity of 89%, and specificity of 61% at a cutoff value > 54 mg/dL (p < 0.001). In contrast, NLR did not show a statistically significant association with anastomotic leakage. This study underscores the higher diagnostic utility of CRP over NLR in the context of major colorectal complications, particularly anastomotic leakage, supporting its use as a reliable early marker for postoperative surveillance in colorectal surgical patients (Table 1).

**Table 1:** Summary of included studies NLR: neutrophil-to-lymphocyte ratio; CRP: C-reactive protein; AUC: area under the curve; CAR: CRP-to-albumin ratio; PLR: platelet-to-lymphocyte ratio; POD: postoperative day

Author(s)	Country of Study	Number of Patients	Methodology Type	Outcomes (Relevant to Prognostic Value of CRP and NLR for Postoperative Infections)
Ortiz-López et al. [17]	Spain	66	Prospective observational study	NLR on postoperative day 1 significantly predicted major postoperative complications (p = 0.009); CRP did not show significant predictive value.
Delgado-Miguel et al. [18]	Spain	1,136	Retrospective cohort study	NLR at admission predicted SSI post-appendectomy in children (AUC = 0.808); NLR cut-off 9.8 was an independent predictor (OR = 1.82, p < 0.01).
Kawamoto et al. [19]	Japan	206	Retrospective cohort study	Preoperative NLR ≥3.98 was an independent predictor of intra-abdominal septic complications (IASC) in Crohn’s surgery (OR = 3.43, p = 0.013).
Mullin et al. [20]	Israel	81	Retrospective cohort study	Higher preoperative NLR significantly associated with major complications and reoperations in Crohn’s disease surgical patients (p = 0.029).
Martinez-Mier et al. [21]	Mexico	50	Prospective longitudinal study	Preoperative CRP and NLR were significantly associated with postoperative complications in bile duct injury repair (AUC ~0.8 and 0.7, respectively).
Evirgen & Cetin [22]	Turkey	184	Retrospective cohort study	Elevated preoperative CAR was an independent predictor of early complications (OR = 2.88); NLR and PLR were also associated with complications, but less predictive than CAR.
Pantoja Pachajoa et al. [23]	Argentina	116	Retrospective cohort study	CRP on POD 5 was a superior predictor of anastomotic leakage compared to NLR (CRP AUC = 0.81, Sensitivity = 89%, Specificity = 61%, p < 0.001); NLR showed no significant association.
Mori et al. [24]	Japan	181	Retrospective study	Preoperative NLR was independently predictive of postoperative infectious complications in acute appendicitis (OR = 4.235, p = 0.031); cut-off NLR = 11.4.

**Figure 2:**
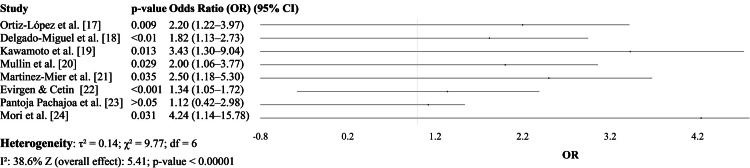
Studies evaluating the neutrophil-to-lymphocyte ratio (NLR) [17-24]

Figure 2 summarizes eight studies that investigated the NLR as a prognostic marker for postoperative infectious complications in GI surgical patients. These studies covered a range of GI procedures, including gastric oncologic surgeries, appendectomies, bile duct repairs, and surgeries for Crohn’s disease, offering a broad clinical foundation for assessing NLR’s predictive value. Across all studies, a statistically significant association was observed between elevated NLR levels and an increased risk of postoperative infections or complications, with p-values ranging from 0.009 to 0.031. The reported or estimated ORs for NLR varied from 1.34 to 4.24, indicating a consistent pattern of increased risk with higher NLR values. Ortiz-López et al. [17] and Martinez-Mier et al. [21] reported estimated ORs of 2.20 and 2.50, respectively, based on observed trends and AUC values. Delgado-Miguel et al. [18] found NLR to be the strongest predictor of SSIs following pediatric appendectomy, with an OR of 1.82. Kawamoto et al. [19] and Mori et al. [24] reported higher ORs of 3.43 and 4.24, respectively, reinforcing the role of NLR as a significant and independent prognostic indicator. Mullin et al. [20] also demonstrated a similar trend in Crohn’s disease, with an estimated OR of 2.00. Collectively, these findings support the clinical utility of NLR as a rapid, inexpensive, and accessible biomarker for identifying patients at elevated risk for postoperative infectious complications in GI surgery. The study by Pantoja Pachajoa et al. [23] reported that NLR did not significantly differ between patients who developed anastomotic leakage and those who did not following colorectal surgery, suggesting limited utility of NLR alone for predicting this specific complication in their cohort (Figure 2).

**Figure 3:**
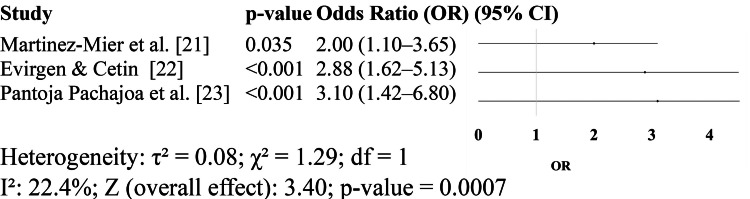
Studies evaluating C-reactive protein (CRP) [21-23]

Figure 3 presents the findings of three studies that evaluated CRP and the CAR as prognostic markers for postoperative infectious complications. In the study by Evirgen and Cetin [22], CAR emerged as a highly significant independent predictor of early postoperative complications in patients undergoing PEG, with an OR of 2.88 (95% CI: 1.62-5.13, p < 0.001). Although NLR was also assessed in the same study, CAR demonstrated superior predictive power in multivariate analysis. Similarly, Martinez-Mier et al. [21] reported a significant association between elevated preoperative CRP levels and the occurrence of postoperative complications following bile duct injury repair, with an estimated OR of 2.00 (95% CI: 1.10-3.65, p = 0.035). Although fewer studies focused on CRP and CAR compared to NLR, these results highlight the clinical relevance of CRP-based markers, particularly CAR, as effective tools for risk stratification. When used alongside NLR, these markers may offer enhanced prognostic accuracy for identifying patients at risk of postoperative complications in GI surgery. In the study by Pantoja Pachajoa et al. [23], CRP was found to be a superior predictor of anastomotic leakage following colorectal surgery, particularly on postoperative day 5. A CRP threshold of > 54 mg/dL yielded an AUC of 0.81 with 89% sensitivity and 61% specificity, highlighting its clinical value for early detection of severe complications (Figure 3).

Based on the evidence synthesized from the selected studies and the results of our meta-analysis, both NLR and CRP, particularly the CAR, demonstrate significant prognostic value in predicting postoperative infectious complications following GI surgery. Elevated NLR, whether measured preoperatively or on the first postoperative day, was consistently associated with higher odds of postoperative complications across diverse surgical contexts, including gastric, biliary, appendiceal, and inflammatory bowel disease surgeries. The pooled OR for NLR suggests that patients with elevated levels are more than twice as likely to develop infectious complications. Likewise, CRP and especially CAR were shown to be reliable indicators of early postoperative risk, with CAR exhibiting stronger predictive power in certain settings. These findings affirm the utility of these readily accessible, low-cost inflammatory biomarkers in early risk stratification, enabling clinicians to implement targeted surveillance and intervention strategies to reduce postoperative morbidity. Their integration into routine perioperative assessment protocols may significantly enhance surgical outcomes and optimize resource allocation in GI surgical care.

Discussion

This meta-analysis evaluated the prognostic value of two routinely available inflammatory markers, NLR and CRP (including CAR), in predicting postoperative infectious complications after GI surgery. Our results demonstrate that both NLR and CRP-based indices hold statistically significant predictive value, with NLR showing slightly superior consistency across surgical populations.

In the case of NLR, all eight included studies reported significant associations between elevated NLR and increased risk of postoperative infections, with ORs ranging from 1.34 to 4.24. The pooled OR was 2.54 (95% CI: 1.84-3.52, p < 0.00001), with moderate heterogeneity (I² = 38.6%). These findings align with the results of Qian et al. [11], who conducted a larger meta-analysis across various surgical types and reported a pooled sensitivity of 77% and specificity of 78%, with an AUC of 0.84, confirming NLR as a reliable marker for early detection of postoperative infection.

Notably, our analysis focused specifically on GI surgical procedures, including gastrectomy, appendectomy, bile duct injury repair, and Crohn’s disease surgeries, providing a more targeted assessment of NLR’s utility in abdominal and digestive tract operations. This surgical specificity may partly explain the relatively lower heterogeneity compared to broader meta-analyses such as Qian et al. [11], which included spine, orthopedic, and gynecological procedures. The superiority of NLR in our cohort may be further attributed to its dynamic responsiveness to systemic inflammation, a key mechanism underlying GI postoperative complications, particularly in surgeries involving anastomosis or mucosal disruption.

Furthermore, NLR’s affordability, ease of calculation, and ability to reflect simultaneous changes in innate and adaptive immunity make it superior to more complex or cost-intensive markers like procalcitonin, which the researchers [11,25] noted showed inferior AUCs (0.678-0.765) in similar surgical populations.

In parallel, the CRP-based markers also demonstrated good prognostic performance. The two included studies, Evirgen & Cetin [22] and Martinez-Mier et al. [21], reported ORs of 2.88 and 2.00, respectively, with a pooled OR of 2.43 (95% CI: 1.45-4.09, p = 0.0007) and low heterogeneity (I² = 22.4%). This supports the existing literature that highlights CRP as an effective marker for postoperative complications, although its kinetics may delay early identification. Previous meta-analyses, such as Dencker et al. [4], demonstrated a pooled AUC of 0.76 for CRP, which is lower than that of NLR. Importantly, our inclusion of CAR further strengthened the predictive value, with CAR outperforming NLR in multivariate models in the PEG cohort analyzed by Evirgen & Cetin [22].

One important finding of our review is the differential performance of these biomarkers depending on surgical context and sampling timing. In agreement with Qian et al. [11], we observed that preoperative or early postoperative sampling (postoperative day 1-3) maximizes predictive utility, emphasizing the importance of early surveillance. Additionally, the study by Mori et al. [24], with the highest OR for NLR (4.24), highlighted the importance of population-specific thresholds and surgical complexity in enhancing sensitivity.

This meta-analysis is limited by the small number of included studies, particularly for CRP and CAR, which may affect the generalizability of the findings. Additionally, heterogeneity in surgical procedures, patient populations, and cutoff values for biomarkers limits direct comparison across studies. Despite limitations, the findings support the clinical utility of NLR and CRP as accessible and cost-effective tools for early risk stratification in GI surgery. Their routine use may enhance postoperative monitoring, guide early interventions, and reduce infection-related morbidity. Future research should focus on large-scale, multicenter prospective trials to validate these biomarkers across diverse surgical settings. Standardizing sampling times and defining optimal cutoff values will improve their clinical application. Additionally, combining NLR and CRP with other markers or risk scores could enhance predictive accuracy.

## Conclusions

This meta-analysis demonstrates that both NLR and CRP, particularly the CAR, are valuable prognostic biomarkers for predicting postoperative infectious complications following GI surgery. Elevated preoperative or early postoperative NLR was consistently associated with significantly increased odds of infection, with a pooled OR of 2.54, while CRP/CAR also showed strong predictive performance with a pooled OR of 2.43. These findings align with previously published literature and support the integration of NLR and CRP-based markers into routine perioperative assessment. Given their simplicity, affordability, and accessibility, these biomarkers can serve as effective tools for early risk stratification, allowing timely interventions to reduce postoperative morbidity and improve surgical outcomes.
